# Eight Weeks of Lifestyle Change: What are the Effects of the Healthy Lifestyle Community Programme (Cohort 1) on Cortisol Awakening Response (CAR) and Perceived Stress?

**DOI:** 10.1177/24705470221099206

**Published:** 2022-09-28

**Authors:** Corinna Anand, Karin Hengst, Reinhold Gellner, Heike Englert

**Affiliations:** 1 Faculty of Medicine, 39069 University of Muenster (WWU), Muenster; 2 Department of Food Nutrition Facilities, 237055 University of Applied Sciences Muenster, Muenster

**Keywords:** cortisol awakening response, perceived stress, healthy lifestyle, controlled intervention, real-world community approach (5-10)

## Abstract

**Background:** Stress and cortisol dysregulation are linked to NCDs. Moreover, stress favours unhealthy lifestyle patterns, which increase the risk for NCDs. The role of the Cortisol Awakening Response (CAR) and the effect of lifestyle interventions on the same remain unclear. **Methods:** The impact of the intensive 8-week phase of the Healthy Lifestyle Community Programme (HLCP, cohort 1) on parameters of the CAR, ie cortisol values 0 (sample [S]1), 30), 45 and 60 minutes post-awakening, average peak, S1-peak delta and area under the increase curve (AUC_I_), and perceived stress levels (PSL) was evaluated in a non-randomized, controlled trial. Covariates of the CAR (eg sleep measures) and irregularities in sampling were assessed. The intervention focussed on stress management, a healthy diet, regular exercise, and social support. Participants were recruited from the general population. Multiple linear regression analyses were conducted. **Results:** 97 participants (age: 56 ± 10 years; 71% female), with 68 in the intervention group (IG; age: 55 ± 8, 77% female) and 29 participants in the control group (CG; age: 59 ± 12, 59% female), were included in the analysis. The baseline characteristics of both groups were comparable, except participants of IG were younger. On average, the PSL at baseline was low in both groups (IG: 9.7 ± 5.4 points; CG: 8.5 ± 6.9 points; p = .165), but 22% (n = 15) in the IG and 20% (n = 6) in the CG reported a high PSL. Most participants reported irregularities in CAR sampling, eg interruption of sleep (IG: 80% CG: 81%). After 8 weeks, most CAR parameters and the PSL decreased in the IG and CG, resulting in no differences of change between the groups. In the IG only, a decrease of PSL was linked to an increase of CAR parameters, eg AUC_I_ (correlation coefficient = −0.307; p = .017). **Conclusion:** The HLCP may potentially reduce PSL and change the CAR, but results cannot be clearly attributed to the programme. Methodological challenges and multiple confounders, limit suitability of the CAR in the context of lifestyle interventions. Other measures (eg hair-cortisol) may give further insights. **Trial registration**: German Clinical Trials Register (DRKS); DRKS00018821; www.drks.de

## Introduction

Stress is the body's ability to adapt and respond to challenging environmental demands.^1^ In modern societies, a majority of citizens report high levels of stress and stress-derived health problems.^2,3^ It has been extensively shown that stress promotes weight gain^4,5^ and increases the risk of noncommunicable diseases (NCDs), including obesity,^6,7^ type 2 diabetes (T2D),^8,9^ hypertension,^10^ the metabolic syndrome (MetS)^11^ and cardiovascular diseases (CVD).^12,13^ Though the underlying mechanisms are not fully understood, it has been established, that stress can affect health directly through neuroendocrine, metabolic and psychobiological pathways, and indirectly through unfavorable changes in health behaviours.^1^

Cortisol plays a key role in the connection of stress and NCDs.^1^ Numerous studies have linked different cortisol parameters to health outcomes: increased a more frequent hypothalamic-pituitary-adrenal (HPA) axis activation activity , elevated cortisol reactivity and cortisol dysregulation have been linked to an increased risk of CVD events,^12,14^ insulin resistance^15^ and T2D,^14,16^ a higher body mass index (BMI), abdominal obesity, and increased systolic blood pressure (SBP).^17,18^ The Cortisol Awakening Response (CAR) is the marked increase in secretion in the first hour immediately post-awakening^19^ and is considered to be representative for the cortisol secretion throughout the day.^20^

Once released, cortisol is involved in immune and inflammatory processes but also in the regulation of metabolic functions, eg it increases access to energy stores, elevates blood sugar levels and increases protein and fat mobilization.^21^ A disruption of its diurnal rhythm is likely to affect these systems and to have an adverse impact on health over time^1^: For instance, cortisol directly promotes fat deposition, particularly in the abdominal region,^22,23^ but a greater BMI predicted less cortisol-mediated inflammation, indicating impaired immune suppressive effects by cortisol in obese individuals.^24^ Results on the CAR and metabolic diseases are controversial: a blunted CAR has been linked to an unfavourable metabolic and cardiovascular risk profile,^25–27^ while others found adiposity indices to not be associated with the CAR.^28^

Also, psychological stress states may be indicated by the CAR^23,29,30–33^, but again, results are inconsistent: a meta-analysis of 147 studies found a *higher* CAR to be linked to psychosocial stress,^34^ while other studies, found a *blunted* CAR in participants with high levels of psychological stress.^35–37^ Hence, different psychosocial factors may have a different effect on the CAR.^34^

Moreover, stress may especially lead to disease in people with too few psychosocial resources,^38^ a lack of social support^39,40^ and poor coping skills.^38^ Stress also favours unhealthy behavioural patterns,^41^ which increase the risk of NCDs.^42,43^ For instance, chronically stressed people, tend to eat more palatable, energy-dense foods that favour weight gain and obesity,^6,44,45^ consume less fruits and vegetables and exercise less,^11,13,46–48^ and a blunted CAR has been linked to poor lifestyle choices.^49^ Additionally, stress may increase sleep deprivation, which is a known risk factor for NCDs,^45^ and poor sleep quality has been associated with diminished CAR.^50,51^

On the bright side, a healthy lifestyle can protect from stress’ negative impact on health: a diet, rich in essential nutrients, is recognized to support mental well-being in the presence of stress.^52^ Physical exercise can improve psychosocial stress states, eg through distraction, experiencing self-efficacy and improved immunological functions,^53^ and Fekedulegn et al suggested, that sufficient leisure time activity protects from dysregulated CAR due to poor sleep quality.^50^

Ironically, it is stress itself, that has been shown to disrupt healthy lifestyle choices^6^ and can therefore even reinforce itself. There is emerging evidence that stress hinders attempts to implement health behaviour.^45^

Thus, the mechanisms connecting chronic stress to NCDs are complex and research on the CAR is inconsistent.

While comprehensive lifestyle intervention programs, including our own,^54,55^ are suitable to induce weight loss, to improve metabolic parameters (eg reduce total- and LDL cholesterol) and meet the complexity of sustainable behaviour change,^56,57^ little is known about the effect of such programmes on the CAR and their potential to reduce stress and its immense burden on health.^45^ Given the complex interaction of direct and indirect influences of stress on health, and considering, that health behaviour change is challenging, *especially* against the background of chronic stress,^45^ holistic approaches are needed to design interventions for the prevention and treatment of NCDs. Moreover, studying the effect of such interventions on the CAR, may provide findings to add to the ongoing discussion about the direction of a possible connection of stress and the CAR in the context of overweight and NCDs.

### Research gap

Stress management interventions can be beneficial for patients with existing NCDs^38,58,59^ but they have not been sufficiently focussed in the prevention of NCDs.^60^ Specifically, data from general-population trials on stress and NCDs and scalable interventions, rather than individual taylor-made solutions, are limited.^61^

The effects of holistic intervention programmes^45^ on the CAR and PSL need to be explored to determine their potential role in the prevention and treatment of stress-related diseases, overweight and NCDs^41,62,63–65^. Moreover, identifying reliable hormonal stress markers to objectify these effects would provide an opportunity for earlier intervention.

### Research Objective

The holistic Healthy Lifestyle Community Program (HLCP) has been shown to significantly reduce weight and parameters of the metabolic risk profile.^54,55^

Here, we examined the effects of the intensive and most effective^55^ 8-week phase of the HLCP (cohort 1) on the CAR hormonal (cortisol, measured in saliva) and psychosocial stress the PSL through the adoption a healthy lifestyle, characterized by 1) a good stress management and healthy coping mechanisms,^6,38^ 2) a healthy diet, rich in essential nutrients,^41,63^ 3) regular physical activity^66^ and 4) social support.^67^

We hypothesized that 1) indicators of the CAR, ie cortisol value at 0 minutes post awakening (S1), the area under the curve with respect to increase (AUC_I_), the mean increase (cortisol maximum), and the baseline-to-peak increase (baseline/peak Δ)^19,68^ changed levels of hormonal , and that 2) PSL would be reduced after participation in the HLCP compared to baseline and compared to the control group (CG).

We further hypothesized that the CAR hormonal, the and PSL as well as their possible changes would be correlated.

## Methods

### Study Design

We conducted a non-randomized, controlled intervention trial with a duration of 24 months, and this report was derived from an analysis after 8 weeks.^55^ The intervention in this study consisted of the Healthy Lifestyle Community Program (HLCP, cohort 1). The CG received no intervention. A primary report on the effect of the HLCP regarding the metabolic risk profile of NCDs in the long-term is reported elsewhere.^55^

### Study Population

#### Sample size

The sample size was calculated for the primary outcome of weight reduction^55^ and six confirmatory tests were planned to prove the effectiveness of the intervention in terms of weight reduction, and to obtain a power of 0.8 at a global significance level of 0.05, we aimed for 92 participants. Exceeding this minimum sample size was tolerated (as described before).^55^ All eligible participants were included into the present secondary analyses, accordingly.

#### Recruitment

Participants of the IG and CG were recruited in rural Germany and from two separate community (“intervention municipality” and “control municipality”). Notably, we included not only participants with overweight or a high stress level but recruited from the general population,^55,69^ and participants self-selected into the intervention. Participants of the CG only knew they were participating in a health study. As with all lifestyle interventions, blinding of participants or instructors to group allocation was not possible.^54^ Staff performing laboratory assessments, however, were unaware of group allocation. The study was registered in the German Clinical Trials Register (DRKS; reference: DRKS00018821; www.drks.de).

#### Inclusion criteria

Participants ≥18 years, who were capable to understand the study content were included, but participants older than 70 years and those on glucocorticoid-medication were excluded from cortisol-related analyses.^19^ The study was conducted in accordance with the Declaration of Helsinki and was approved by the ethics committee of the Westphalia-Lippe Medical Association and the Muenster University (Muenster, Germany; reference: 2017-105-f-S; approved 5 April 2017). All participants provided written informed consent.

#### Participants’ flow diagram

**Figure 1.**
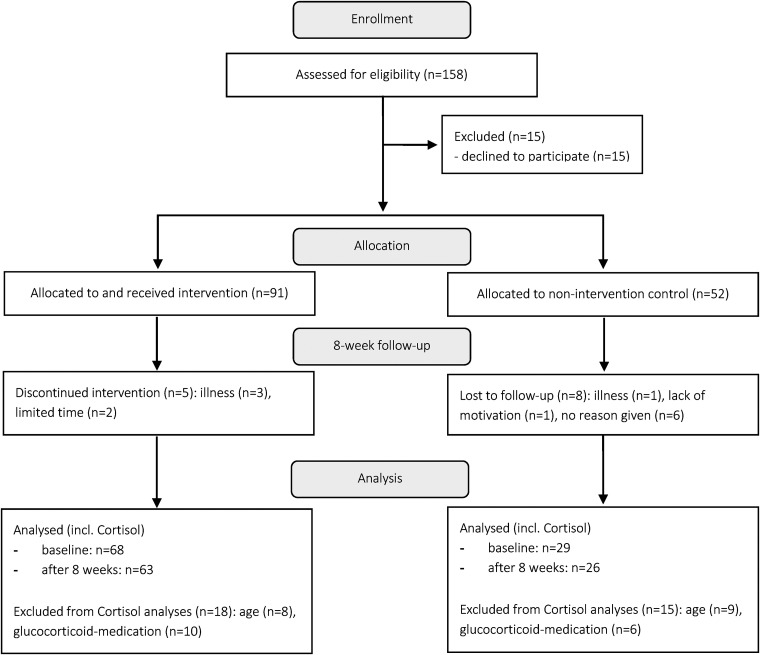
CONSORT structure participants’ flow diagram; participants categorized as “lost to follow-up” withdraw from the study with the given reason. In the IG, information is given on how many participants discontinued the intervention (eg dropped out) and why.

A participants’ flow diagram shows the study process from enrolment to analysis in Figure 1. At baseline, 91 participants were included in IG and 52 in CG. In total, 97 participants (age: 56 ± 10 years; 71% female) met the inclusion criteria for the analyses of the CAR, with 68 in the IG and 29 in the CG (see Table 1).

**Table 1.** Baseline characteristics by study group (n = 97).

	INTERVENTION	CONTROL	p-value
	n = 68	n = 29	
**SOCIODEMOGRAPHICS**			
**age**, mean ± SD	55 ± 8	59 ± 12	**.009** ^1^
**female**, n (%)	52 (77)	17 (59)	.090^2^
**ANTHROPOMETRICS**			
**BMI**, mean ± SD	28.5 ± 5.3	27.8 ± 7.3	.251^1^
**HORMONAL STRESS PARAMETERS**			
**cortisol** (nmol/l)			
**min upon waking**^3^, mean ± SD			
0 min	9.97 ± 7.42	10.61 ± 4.57	.132^1^
30 min	14.83 ± 5.75	15.96 ± 6.80	.488^1^
45 min	15.54 ± 11.08	15.66 ± 7.18	.584^1^
60 min	13.72 ± 11.77	12.83 ± 4.95	.747^1^
**Cortisol maximum**^3^, mean ± SD	17.47 ± 11.85	17.73 ± 7.43	.179^1^
**time of maximum**, n (%)			
0 min	8 (12)	5 (17)	.521^2^
30 min	33 (49)	15 (52)	.827^2^
45 min	18 (27)	6 (21)	.796^2^
60 min	9 (13)	3 (10)	1.000^2^
**baseline/peak** Δ ^3^, mean ± SD	7.50 ± 7.14	7.13 ± 7.47	.559^1^
**AUC_I_**^3^, mean ± SD	221.21 ± 235.23	217.32 ± 287.15	.646^1^
**COVARIATES OF THE CAR**			
**Sampling irregularities** ^3^	n = 21	n = 6	** **
exercise evening before, n (%)	0	0	1.000^2^
insomnia, n (%)	9 (15)	3 (11)	.508^2^
delayed sampling, n (%)	4 (7)	1 (4)	1.000^2^
breakfast during sampling, n (%)	1 (2)	0	1.000^2^
sleep between samples, n (%)	1 (2)	0	1.000^2^
other irregularities, n (%)	6 (10)	2 (7)	.492^2^
**Sleep measures** ^3^			** **
falling asleep (hours), mean ± SD	22:14 ± 2:03	20:32 ± 5:23	.898^1^
awakening (hours), mean ± SD	6:00 ± 0:40	6:35 ± 0:41	**<.001** ^1^
intermediate waking times, n (%)	n = 61	n = 29	.178^2^
never	3 (5)	3 (10)	
once	20 (33)	4 (15)	
twice	22 (36)	10 (35)	
three times	7 (11)	9 (31)	
four times	6 (10)	3 (10^4^)	
five times or more	3 (5)	0	
**stressful day ahead anticipated^3^**	n = 57	n = 28	
yes, n (%)	10 (18)	8 (29)	.444^2^
**perceived socioeconomic status^3^**	n = 61	n = 29	
low, n (%)	1 (2)	0	.083^2^
medium, n (%)	49 (80)	18 (63)	
high, n (%)	11 (18)	22 (37)	
**PSYCHOSOCIAL STRESS PARAMETERS**			
**Perceived stress**, mean ± SD	9.7 ± 5.4	8.5 ± 6.9	.165^1^
high stress level^7^, n (%)	15 (22)	6 (20)	.523^2^

min = minutes, AUC_I_ = area under the curve with respect to increase, CAR = Cortisol Awakening Response.

^1^ Mann-Whitney-U test, ^2^ Fisher's exact test, ^3^ average of day 1 + 2, ^4^ total percentages exceed 100 due to rounding up ^5^ <5 drinks/month ^6^ ≥ 2 drinks/week, ^7^ PSS-10 Score^49^ ≥ 14.

### Lifestyle Intervention

The intervention has been described in detail previously.^55^ In short, the 8-week intensive phase of the comprehensive HLCP consisted of 14 consecutive seminars (2x/week for 2 hours each) with a strong emphasis on salutogenesis and how to use personal health resources than the chances of behaviour change . Stress management was one of four main topics, next to a healthy, predominantly plant-based diet, physical activity and social health,^55^ and one of the seminars was exclusively about stress as a health factor. Mini-mindfulness exercises (eg ‘take one minute to think about what would make you feel good’) were integrated into all sessions. Stress workshops (eg ‘relaxation techniques’) were offered in smaller groups (∼20 participants each; ∼2-hour duration) to allow for more room for individual coping mechanisms and to strengthen group support. Moreover, each participant received two individual health coachings, in which results of the health checks, personal goals and recommendations for lifestyle changes were discussed. In a 22-months-follow-up-phase, monthly refresher-meetings (2 hours each) repeated and deepened content and strengthened group support.

Participants of the CG did not receive any intervention, but were informed about health checks results (ie they could seek medical advice if needed) for ethical reasons and were offered to participate in a subsequent HLCP after completion of the study.

### Data Assessment

Baseline data were collected in April and October 2017 in IG and CG, respectively (see,^55^) and all measures were equivalently taken in both groups after 8 weeks. Since funding was provided at relatively short notice, time and staff unfortunately were insufficient to recruit and start both study arms at the same time.

CAR and PSL were assessed besides general parameters from the main study,^55^ which included sociodemographic characteristics (eg age and sex), anthropometric parameters (eg body mass index; BMI), blood parameters (eg fasting glucose, insulin, lipids), and vital parameters (eg blood pressure) as well as dietary and health behaviour among others.

#### Cortisol awakening response (CAR)

The hormonal stress status was assessed by salivary cortisol measurement in order to determine the Cortisol Awakening Response (CAR), which is the marked increase in cortisol secretion in the first hour immediately post-awakening. ^19^ It is considered to be representative for the cortisol secretion throughout the day. 20

CAR was measured in saliva. At baseline and 8 weeks, saliva was collected at home by the participants, on two subsequent days upon waking (0 minutes [min]; sample 1; S1) as well as at 30, 45- and 60-min post-wakening (samples S2-S4). The samples were collected using Salivettes® (Sarstedt, Germany). Participants chewed a swab gently for one minute in order to collect saliva, and then deposited it into the provided sample tube. The samples were stored in the participants’ refrigerators and collected in the intervention venue by the study team within the next day.

Samples were taken on two weekdays (no weekend or holiday^20^) and their mean was calculated. Participants were asked to refrain from drinking water before the first sample. During the whole sampling time they were also instructed to refrain from brushing their teeth, smoking, eating, drinking coffee, and exercising (also the evening before) and to report all kinds of irregularities (eg delayed sampling). Several covariates and possible confounders of the CAR^19^ were assessed: self-reported sleep measures (ie time of awakening and falling asleep, frequency of intermediate waking times), anticipation of a stressful day ahead, perceived socioeconomic status, smoking habits, alcohol consumption, and oral contraceptive use. As the study documents were already very extensive, covariates were reported via self-report in a simple non-validated questionnaire.

To ensure high data quality and compliance,^30^ participants were extensively informed about the measurement procedures, in person and with a detailed, easy-to-understand flyer. Questions were answered with great care and patience.

The samples were analysed using a Competitive Enzyme Linked Immunosorbent Assay (ELISA) kit (daacro Saliva Lab Trier, Germany).

To capture the dynamic of post-awakening cortisol changes (CAR), the area under the curve with respect to increase (AUC_I_), the mean increase, and the baseline-to-peak increase (baseline/peak Δ) were assessed.^19^

#### Perceived stress level (PSL)

Participants completed the German version of the Perceived Stress Scale-10 (PSS-10),^70^ which is a reliable tool (Crohnbachs Alpha [α] =  0.78) to assess the PSL of the past 30 days.^71,72^ Each item (eg “In the last month, how often have you felt that you were unable to control the important things in your life?”) was rated on a 5-point Likert scale ranging from 0 (never) to 4 (very often). The respective sum score (ranging from 0 to 40) was calculated, with higher scores indicating higher PSL. In order to compare high stress versus low stress, participants who scored ≥14 on the PSS were allocated to the high-stress group.^49,71^

### Statistics

All data were analysed according to a predefined plan and all available cases were used. Missing data were not imputed. Continuous variables are presented as mean ± standard deviation (SD), categorical variables as frequencies and valid percent. Normality was tested using the Shapiro–Wilk test and judged by histograms.

To compare IG and CG, independent t-test was used for normally distributed continuous variables and Mann–Whitney-U test as its nonparametric alternative. Comparisons of dichotomous categorical variables were tested by Fisher's exact test. Within-group comparisons for normally distributed continuous variables were performed by one-sample *t-*test. Alternatively, the nonparametric Wilcoxon signed-rank test was performed. Relations between two continuous variables, eg the hypothesized connection of the changes of the CAR and the PSL were assessed with Spearman correlation analyses. All tests were two-sided.

Cortisol values were log-transformed to attain normality.^19^ However, estimates and standard errors were back-transformed to aid interpretability of the results.^73^ As the CAR was assessed on two separate days, the mean of the values was calculated and used for further analyses. In case, one value was missing, the value of the other day was used instead of the mean. If both values of a time point were not available, they were treated as a missing value and not replaced.

Sensitivity analyses were conducted, excluding cases and those with other irregularities (eg insomnia or delayed sampling) as well as outliers.

Multiple linear regression (MLR) models were used to estimate the effect of the intervention on the change of 1) the CAR, ie S1, AUC_I_, the mean increase, baseline/peak Δ. The respective baseline values, sex, age, BMI, sleep measures, anticipation of a stressful day ahead, perceived socioeconomic status, smoking habits, alcohol consumption, and oral contraceptive use^19^ were first examined in separate univariate used as covariates in the regression models to determine account for their known effect on the CAR using a forced enter selection approach.

Similarly, the effect of the HLCP on the change of 2) the PSL has been investigated in MLR models, adjusting for the respective baseline values, sex, age. Residuals of all models have been checked for normality.

p-values <.05 are considered significant, but are to be understood exploratory^74^ as all presented data are secondary outcome parameters.

Statistical analyses were performed using IBM SPSS Statistics for Windows, Version 27.0. Armonk, NY: IBM Corp.

## Results

Baseline characteristics of IG and CG are shown in Table 1. We observed no significant differences in most parameters, but participants of the IG were younger (p = .009) and averagely awoke earlier in the morning (p <.001).

### CAR and PSL at Baseline

The maximum of cortisol values was 17.47 ± 11.85 nmol/l in the IG and 17.73 ± 7.43 nmol/l in the CG, respectively, and the peak was reached after 30 minutes in most participants (see Table 1). In both groups, very less participants reported no interruption of sleep the nights before sampling and most of them (IG: 80%, CG: 81%) woke up one to three times.

Both groups combined, 19 participants at baseline and 12 participants after 8 weeks (ie approximately one fifth of participants) had a negative AUC_I_, which means that cortisol did not increase after S1. This indicates that the peak was reached before awakening (possibly due to waking up at night) or that sampling was delayed. In order to conduct sensitivity analyses we excluded these cases and those with other irregularities (eg insomnia, see Table 1) as well as one outlier (n = 1), resulting in only n = 40 (45%) complete cases out of 89 cases, that were eligible for CAR analyses. However, the sensitivity analyses revealed no significant differences in any of the cortisol markers (ie cortisol values of S1-4, average peak value, time of the peak, baseline-peak Δ and AUC_I_).

The PSS-10 score was comparable in both groups at baseline with 9.7 ± 5.4 in the IG and 8.5 ± 6.9 in the CG (p = .165), indicating a low average PSL. Yet, one fifth of the participants (22% in the IG and 20% in the CG) reported a high PSL (Table 1).

Highly stressed participants (PSS-10 ≥ 14 points) of both groups combined (n = 32) had a higher BMI (31 ± 8 kg/m^2^^)^ than those with a low PSL (27 ± 6 kg/m^2^; p = .006) and were more often overweight or obese (84% [n = 27] vs. 62% [n = 68]; p = .020).

### Changes After 8 Weeks

**Figure 2.**
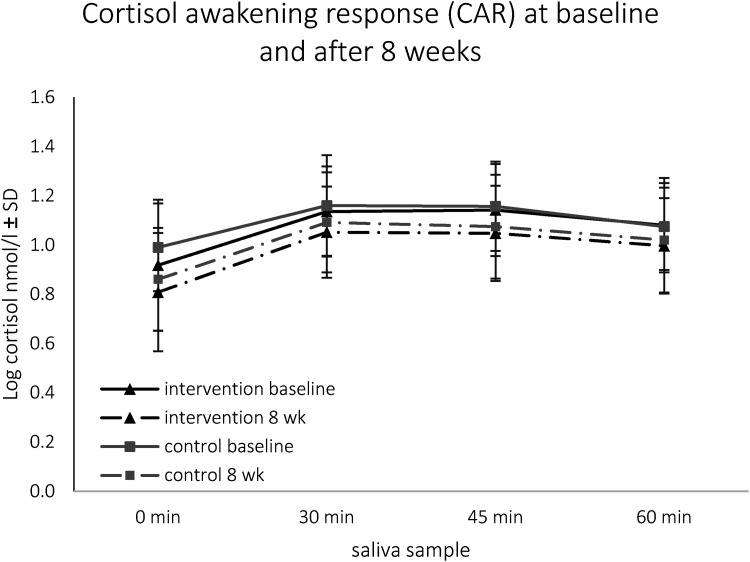
Log transformed salivary cortisol (nmol/l) sampled 0, 30, 45 and 60 minutes (min) upon awakening at baseline and after 8 weeks (wk) in intervention and control group. Lower cortisol levels after 8 weeks in the intervention group at 0 min (p = .002), 30 min (p = .005), 45 min (p = .001) and 60 min (0.015) and in the control group at 0 min (p = .004), 30 min (p = .009), 45 min (p = .035) and 60 min (p = .020).

Average cortisol levels of samples S1 - S4 decreased after 8 weeks in the IG, the same was true for the CG (see Figure 2), resulting in no significant differences between the groups. Also, the cortisol maximum and the baseline-peak Δ followed this pattern and were lower in both groups, with no significant differences between IG and CG (see Table 2). The change of the AUC_I_ after 8 weeks, however, was non-significant in both groups, again resulting in no significant differences between IG and CG. The results were confirmed in sensitivity analyses including only participants without irregularities.

**Table 2.** Stress Parameters in the Intervention and Control Group Before and After the Intervention (Means ± SD).

	Comparison of change								
	within group								between group
	**INTERVENTION GROUP**				**CONTROL GROUP**				
	**Before**	**after**	**Change**	**p-value**	**before**	**after**	**change**	**p-value**	**p-value**
**cortisol (nmol/l)**	n = 68	n = 63			n = 29	n = 26			
**min upon waking^3^**									
0 min	10.0 ± 7.4	7.5 ± 4.3	−2.6 ± 7.4	**.001**	10.61 ± 4.57	8.2 ± 4.2	−2.9 ± 3.8	**<.001**	.303
30 min	14.8 ± 5.8	12.3 ± 4.9	−2.6 ± 5.3	**<.001**	15.96 ± 6.80	13.6 ± 6.3	−2.3 ± 5.1	**.043**	.760
45 min	15.5 ± 11.1	12.2 ± 4.8	−3.6 ± 10.7	**<.001**	15.66 ± 7.18	13.1 ± 6.3	−2.9 ± 6.1	**.030**	.728
60 min	13.7 ± 11.8	10.8 ± 4.3	−3.1 ± 11.6	**.001**	12.83 ± 4.95	11.5 ± 5.4	−1.6 ± 5.3	.182	.704
**cortisol maximum^3^**	17.5 ± 11.9	14.0 ± 5.1	−3.7 ± 11.5	**<.001**	17.73 ± 7.43	15.8 ± 6.6	−2.6 ± 5.5	**.028**	.889
**baseline/peak Δ ^3^**	7.5 ± 7.1	14.4 ± 9.1	6.8 ± 8.1	**<.001**	7.13 ± 7.47	15.1 ± 10.8	8.3 ± 8.1	**<.001**	.626
**AUC_I_^3^**	221.2 ± 235.2	204.7 ± 240.7	−17.0 ± 241.5	.664	217.32 ± 287.15	221.1 ± 296.7	+ 30.1 ± 220.4	.367	.354
**Perceived stress level**	9.7 ± 5.4	6.2 ± 4.8	−3.6 ± 4.8	**<.001**	8.5 ± 6.9	5.9 ± 6.1	−2.9 ± 3.4	**<.001**	.989

The PSS-10 Score dropped noticeably in both IG and CG by −3.6 ± 4.8 (−36%; p <.001) and −2.9 ± 3.4 (−31%; p <.001), respectively, once more resulting in no difference between the two groups (p = .989).

### Bivariate Correlations

As Table 3 shows, S1 at baseline was negatively correlated to the time of awakening and positively correlated to the PSL, indicating that a higher cortisol value upon wakening was linked to early awakening and a higher PSL. None of the cortisol parameters were correlated to the other baseline characteristics, including BMI and other known covariates.^19^

**Table 3.** Bivariate Correlations Between Baseline Stress Parameters and Other Baseline Characteristics (Intervention and Control Group Combined).

			STRESS PARAMETERS								
	cortisol S1		cortisol AUC_I_		cortisol maximum		cortisol baseline/peak Δ		PSS-10		
	cc	p-value	cc	p-value	cc	p-value	Cc	p-value	cc	p-value	n
**Age**	0.133	.196	−0.134	.344	0.045	.751	−0.094	.504	−0.330	**.001**	97
**BMI**	−0.007	.942	−0.114	.267	−0.079	.444	−0.110	.285	0.195	.054	98
**time of falling asleep**	−0.048	.657	−0.054	.706	0.065	.642	0.197	.084	−0.142	.179	91
**awakening time**	0.266	**.032**	−0.026	.853	0.225	.106	−0.049	.670	−0.098	.357	78
**intermediate waking**	−0.164	.124	0.190	.178	0.101	.470	−0.066	.552	−0.102	.321	84
**stressful day anticipated**	−0.002	.987	−0.161	.256	−0.112	.423	−0.134	.223	−0.074	.471	84
**perceived socioeconomic status**	0.035	.741	−0.011	.939	0.101	.472	−0.033	.774	−0.261	**.013**	77
**perceived stress level**	0.260	**.013**	−0.062	.660	−0.091	.518	−0.062	.575	–	–	84

AUC_I_ = area under the curve with respect to increase; PSS-10 = perceives stress scale [Quelle]; cc = Spearman correlation coefficient.

As described before, the weight as the primary outcome decreased significantly after 8 weeks,^55^ but we detected no link of this decrease to changes of any of the CAR parameters (S1: cc = 0.046, p = .727; cortisol maximum: cc = 0.213, p = .103; baseline-peak Δ: cc = 0.126, p = .338; AUC_I_: cc = 0.142, p = .280) or the change of PSL (cc = 0.065, p = .572).

Notably, in the IG but not in the CG, the change of PSL was negatively correlated to the baseline-peak Δ (cc = −0.384; p = .002) and the AUC_I_ (cc = −0.307; p = .017), indicating that a reduction of the PSL was linked to an increase of the CAR parameters.

### Regression Analysis

The group affiliation was first examined in separate univariate multiple regression models to determine their effect on the changes of the CAR and PSL after 8 weeks. According to our observation of no group differences regarding any cortisol parameter, the variable *group* neither predicted the change of any of the cortisol parameters nor of the PSL (see Table 4).

**Table 4.** Effect of Group (ref. Intervention Group) on the Changes of CAR Parameters (Adjusted for sex, Baseline Values Adjusted for sex, age, BMI, Sleep Measures, Anticipation of a Stressful day Ahead, Perceived Socioeconomic status, Smoking Habits, Alcohol Consumption, and Oral Contraceptive use; Forced Enter Approach).

	Effect of the intervention on changes after 8 weeks			
	ß	SE	p =	R^2^
**CAR parameter**				
S1	−0.93	1.82	.609	0.116
cortisol maximum	2.46	2.93	.404	0.037
baseline-peak Δ	1.51	2.43	.535	0.035
AUCI	45.94	68.44	.504	0.045
**Perceived stress level**	0.75	1.01	.462	−0.005

*C*AR = Cortisol Awakening Response; ß = effect size for group; SE = standard error; R^2^ = corrected R^2^; S1 = cortisol sample 1 immediately after awakening (0 min) Constants (ß_0_) and its SE are not shown and numbers are rounded to two decimals to improve readability.

## Discussion

This study explored the beneficial effects of the holistic Healthy Lifestyle Community Program (HLCP, cohort 1) on the Cortisol Awakening Response (CAR) and perceived stress level (PSL).

### Cortisol Awakening Response (CAR)Hormonal Stress Status

All parameters of the CAR, except the AUC_I_, decreased after 8 weeks of the HLCP, and the same was true for the control group (CG).

Hence, our results underline, what O’Connor et al (2021) conclude: Stress research ‘moved away from a simple model according to which stress would result in too much cortisol […] and towards an understanding that [different] systems interact and become dysregulated in more nuanced ways.’ ^1^ To capture these nuances, more measurements and a larger study cohort may give better insights on the effects of the HLCP on hormonal stress levels.

Moreover, However, the immense amount of possible covariates^19^ challenge the comparability of results, which are also prone to be influenced by irregularities. Several covariates that might have influenced the CAR were not assessable (eg ambient light level,^75^^)^ others, like delayed sampling, were assessed via self- disclosure. Here, only very few participants (at baseline IG: 7%, CG: 4%) stated a delay. Yet, we observed negative AUC_I_s in one fifth of the participants, which possibly indicate delayed sampling.^19^ As a delay of 5 to 10 minutes can already impair CAR results,^19^ future research in the real-world setting should use new technologies (eg electronic containers that record the time of sampling^1^^)^ to assess delays more accurately.

Moreover, when excluding cases with a possible un-reported delay and those who self-reported at least one irregularity (eg insomnia, delayed sampling), a mere 45% of the cases in our study could be considered complete. Though sensitivity analyses did not reveal significant differences, undetected irregularities and unassessed confounders may have influenced the results of our investigation.

Cortisol levels typically follow a strong diurnal rhythm, with levels increasing before and co-initiating awakening, being highest within the first hour after awakening, dropping rapidly in subsequent few hours and then dropping more slowly until reaching a nadir around bedtime.^76,77^ CAR is extremely dependent on being determined at the right time time of day accounts for up to 72% of the variance in salivary cortisol levels, ^78^ and even though we educated the participants extensively about the method and did assess the timing of sampling, we could not verify these information, as participants took the samples at home. Also, the diurnal cortisol rhythm varies from day to day and individual differences have to be accounted for. ^77^ A study of healthy volunteers (N = 15) in the sleep laboratory indicates that cortisol secretion immediately after waking and the extent of the cortisol increase react sensitively to different daily activities and vary accordingly.

As CAR assessment has been shown to be highly influenced by situational factors, 2–6 days of assessment on work days are needed.^79^ In our study, the minimum of two days of sampling^19^ was chosen due to practical and financial reasons. However, sampling of more days may improve reliability. For example, Daubenmier et al (2011) sampled for four days and documented significant reduction of the CAR after a mindfulness intervention (n = 19) as well as associations of changes of CAR and abdominal fat.^23^ Our study, though, did not show any link between the significant weight change^55^ and the CAR.

We found however, that the change of PSL after 8 weeks was negatively associated with an increase of the baseline-peak Δ and an increase of the AUC_I_ of the CAR, in the IG and not the CG. Results of other studies regarding the link between psychological stress and the CAR are mixed: Some studies report an increase of the CAR along with an increase of psychological distress, while others suggest that chronic stress may disrupt HPA axis regulation and lead to a blunted CAR,^1^ and a comprehensive meta-analysis concludes, that different psychosocial factors are associated with both enhanced and reduced cortisol awakening responses.^34^

While results on the CAR are inconsistent, hair cortisol has been consistently associated with chronic stress, obesity and NCDs, for example cardiovascular disease, recent myocardial infarction, and Diabetes Mellitus (DM)^16,28,80–83^. The measure is less prone to methodological errors^16,80^ and due to its non-invasive sampling, being independent of the time of day, it may be a reliable indicator in studying the effects of chronic stress on health.^82^

#### Perceived stress level (PSL)

Our study confirms the assumption, that higher PSL is linked to overweight and obesity. However, the significant weight reduction in the IG,^55^ was not associated with the change of PSL. Moreover, we did not find a clear impact of the HLCP on PSL, as it decreased in both groups by more than 30%. Even though the participants of both groups started with relatively low baseline PSL, the drop is comparable with results of a study with a highly stressed cohort. Here, the PSL decreased by 30% after an eight-week stress reduction intervention (from 27.21 ± 7.65 to 19.00 ± 6.44, p = .001).^84^

Yet, we expected differences in the changes of PSL between the IG and CG, which we did not detect. Possibly, receiving their health checks results and being in contact with the study team,^55^ resulted in lower PSL in the CG due to the monitoring of their individual health status. Junne et al (2017) suggest, that PS is determined by a feeling of ineffectiveness, among others, and found self-monitoring of behavioural outcome to significantly improve this ‘ineffectiveness’.^85^ This may thereby have contributed to the PS lowering effect of being int the CG. Moreover, Whitehead and Blaxton (2021) indicate that, on a daily basis, better perceived health is linked to lower PSL. It seems possible, that the perception of health changed in the CG due to the mere participation in our study, resulting in lower PSL.

### Limitations

Our study has three main limitations. Firstly, the results derived from a secondary analysis of a study investigating the primary outcome of weight reduction. As the sample size was not calculated on the basis of expected changes regarding the CAR or PSL, the number of available cases was rather small to study these outcome parameters. Moreover, inclusion criteria did not cover the stress parameters and the average PSL was low. Hence, the results should be confirmed in further studies with a larger sample size and a cohort with a higher baseline PSL. Secondly, the CAR and PSS-10, are parameters to assess a rather acute phases of stress and additional assessment of chronic stress marker would provide valuable information. Thirdly, as season has been shown to influence the CAR,^35^ it is a limitation of our study, that data in the IG and CG were collected six months apart from each other, which might have impaired comparability of the groups.

## Conclusion

The HLCP potentially affects the cortisol-awakening response (CAR) and perceived stress levels, but results cannot be clearly attributed to the programme. Determination of the CAR may not be a reliable hormonal marker to quantify and objectify levels of stress in the context of lifestyle interventions in a real-world, ie non-laboratory, environment, due to confounders and methodological challenges. Measuring hair-cortisol as a cumulative marker for cortisol, and a larger sample with higher baseline stress-levels may give valuable insights on the effect of the HLCP on stress, the relation of chronic stress and NCDs and potential prevention strategies.
